# Comparative Effects of Cytokinins and Spermidine on In Vitro Organogenesis of *Ruscus hypoglossum* L. and Rooting of Regenerated Shoots

**DOI:** 10.3390/plants15172615

**Published:** 2026-08-27

**Authors:** İbrahim Halil Hatipoğlu, Ümmü Özgül Karagüzel

**Affiliations:** Department of Horticulture, Faculty of Agriculture, Recep Tayyip Erdogan University, 53300 Rize, Türkiye; ozgul.karaguzel@erdogan.edu.tr

**Keywords:** cut foliage, kinetin, micropropagation, polyamines, rhizome explant, thidazuron, zeatin

## Abstract

*Ruscus hypoglossum* L. is an evergreen ornamental species valued as cut foliage, but its commercial production is constrained by slow vegetative propagation and limited availability of uniform planting material. This study aimed to develop an efficient in vitro propagation protocol for *R. hypoglossum* by evaluating the effects of different cytokinins and spermidine on shoot organogenesis and subsequent rooting. Thidiazuron (TDZ), zeatin, kinetin, and spermidine were applied at 0.5 and 1.0 mg L^−1^ to rhizome explants derived from in vitro-grown seedlings. Explant length, shoot and cladode number, fresh weight, shoot diameter, chlorophyll a and b, total carotenoid content, SPAD value, and rooting performance were assessed. Significant treatment effects were observed for most traits. Kinetin at 1.0 mg L^−1^ produced the highest shoot proliferation (4.66 shoots explant^−1^), cladode number (12.00 explant^−1^), fresh weight (0.31 g), chlorophyll a (0.72 mg g^−1^ FW), chlorophyll b (0.29 mg g^−1^ FW), and SPAD value (40.25). Spermidine at 1.0 mg L^−1^ resulted in the highest carotenoid content (0.59 mg g^−1^ FW) and promoted greater explant elongation, whereas TDZ at 1.0 mg L^−1^ reduced cladode formation and pigment accumulation. Multivariate analyses consistently identified kinetin as the most favorable treatment during multiplication. Shoots regenerated with 1.0 mg L^−1^ kinetin also showed the highest rooting response following a 10 s pulse treatment with 2.0 mg L^−1^ IBA and transfer to hormone-free half-strength MS medium. Overall, kinetin was the most effective treatment under the conditions tested, providing a practical basis for efficient clonal propagation and ex situ conservation of *R. hypoglossum*.

## 1. Introduction

The ornamental plant industry has expanded considerably in recent years, driven by increasing demand for cut flowers, potted plants, and cut foliage for landscaping, interior decoration, and floriculture [1]. Among these product groups, cut foliage has gained particular commercial importance owing to its decorative value, durability, and ability to maintain its visual quality in floral arrangements [2,3,4,5]. Global production and trade data indicate that ornamental plants constitute a substantial international market, with cut foliage representing a steadily expanding sector supported by increasing demand in both temperate and tropical production regions [6,7,8]. Countries such as the Netherlands, Costa Rica, China, Japan, and India are among the leading producers and exporters of cut foliage, highlighting the growing economic importance of this product group worldwide [9,10].

Within this expanding market, species combining prolonged postharvest longevity with distinctive foliage characteristics are receiving increasing attention as alternative cut-foliage crops. The genus *Ruscus* comprises evergreen, rhizomatous monocots characterized by dark-green, leaf-like cladodes that perform the primary photosynthetic function and retain their ornamental quality for extended periods [11,12]. Among them, *Ruscus hypoglossum* L. is an evergreen perennial species native to southeastern Europe [13]. In Türkiye, it occurs naturally in humid, shaded broad-leaved forests, particularly in the Black Sea region [14]. Owing to its attractive cladodes, extended vase life, and adaptation to local ecological conditions, *R. hypoglossum* represents a valuable native genetic resource with considerable potential for diversification of the cut-foliage industry [15].

Conventional propagation of *Ruscus* relies primarily on seeds, rhizome division, and vegetative propagation. However, these approaches are constrained by slow multiplication rates, seedling variability, and the limited number of propagules obtainable from individual stock plants, which restrict the rapid production of uniform planting material. Consequently, in vitro culture offers a suitable alternative for the controlled and efficient clonal propagation of selected *Ruscus* material [16,17]. Consequently, in vitro propagation has emerged as an efficient alternative for producing genetically uniform planting material while simultaneously supporting ex situ germplasm conservation and reducing harvesting pressure on natural populations [17].

Several regeneration protocols have been reported for *Ruscus* species. Early studies demonstrated successful shoot regeneration of *R. hypophyllum* using liquid induction media and shoot-tip explants [18,19], whereas subsequent investigations optimized micropropagation systems for *R. hypophyllum* and *R. aculeatus* through modifications of culture conditions and multiplication media [20,21,22]. In *R. hypoglossum*, indirect regeneration through callus cultures has been achieved [23], while improvements in seed germination and establishment have also been reported [24,25]. Nevertheless, regeneration efficiency remains highly dependent on explant source, culture conditions, and plant growth regulator composition, indicating that further optimization is still required for reliable large-scale propagation.

Among plant growth regulators, cytokinins play a pivotal role in regulating cell division, shoot induction, and axillary bud proliferation during in vitro culture [26]. Thidiazuron (TDZ) is recognized as one of the most potent synthetic cytokinins for inducing adventitious shoot formation in numerous species [27]. In contrast, naturally occurring or adenine-type cytokinins such as zeatin and kinetin frequently promote shoot elongation and improve shoot quality, although their effectiveness is species dependent [28,29]. Besides cytokinins, polyamines have received increasing attention as regulators of cell division, morphogenesis, organogenesis, and plant responses to environmental stresses. However, their effects on in vitro growth and regeneration may be either promotive or inhibitory depending on the plant species, explant type, polyamine type, and applied concentration. Polyamines are low-molecular-weight polycationic compounds that play a role in various developmental processes, including cell division, differentiation, organ formation, and plant regeneration. Among these, spermidine has attracted particular attention in plant tissue culture due to its role in morphogenetic capacity and its interactions with endogenous hormonal signaling. Exogenous spermidine has been reported to enhance regeneration and micropropagation responses in various plant species, but its efficacy is largely dependent on species, explant type, and concentration [30]. Therefore, spermidine was included in this study as a non-cytokine biologically active regulator to determine whether it can improve shoot organogenesis and growth in *R. hypoglossum* and to compare its effects with those of conventional cytokinin treatments. Spermidine, one of the major endogenous polyamines, has been shown to enhance shoot regeneration, rooting, and stress tolerance in various plant species, with its effectiveness varying according to genotype, explant type, and concentration [31].

Despite previous reports on in vitro regeneration in *Ruscus*, comparative information on the effects of structurally different cytokinins and polyamines on the organogenic response of *R. hypoglossum* is limited. In particular, TDZ, zeatin, kinetin, and spermidine have not been systematically compared under the same culture conditions using explants obtained from the rhizome. Therefore, this study aimed to develop an efficient and reproducible in vitro propagation protocol for *R. hypoglossum* collected from Arhavi (Artvin) in the Eastern Black Sea region of Türkiye, by comparatively evaluating TDZ, zeatin, kinetin, and spermidine at concentrations of 0.5 and 1.0 mg L^−1^ during the propagation stage. The effects of these substances on the multivariate relationships between shoot and cladode development, photosynthetic pigment content, and growth characteristics were evaluated. In addition, the rooting ability of regenerated shoots after IBA pulse treatment was also assessed. This approach aims to determine the most suitable regulatory treatment for efficient shoot propagation and subsequent seedling production, thereby providing a practical basis for clonal propagation and supporting the conservation of this commercially valuable ornamental plant species.

## 2. Results

### 2.1. Morphological Responses

**Figure 1:**
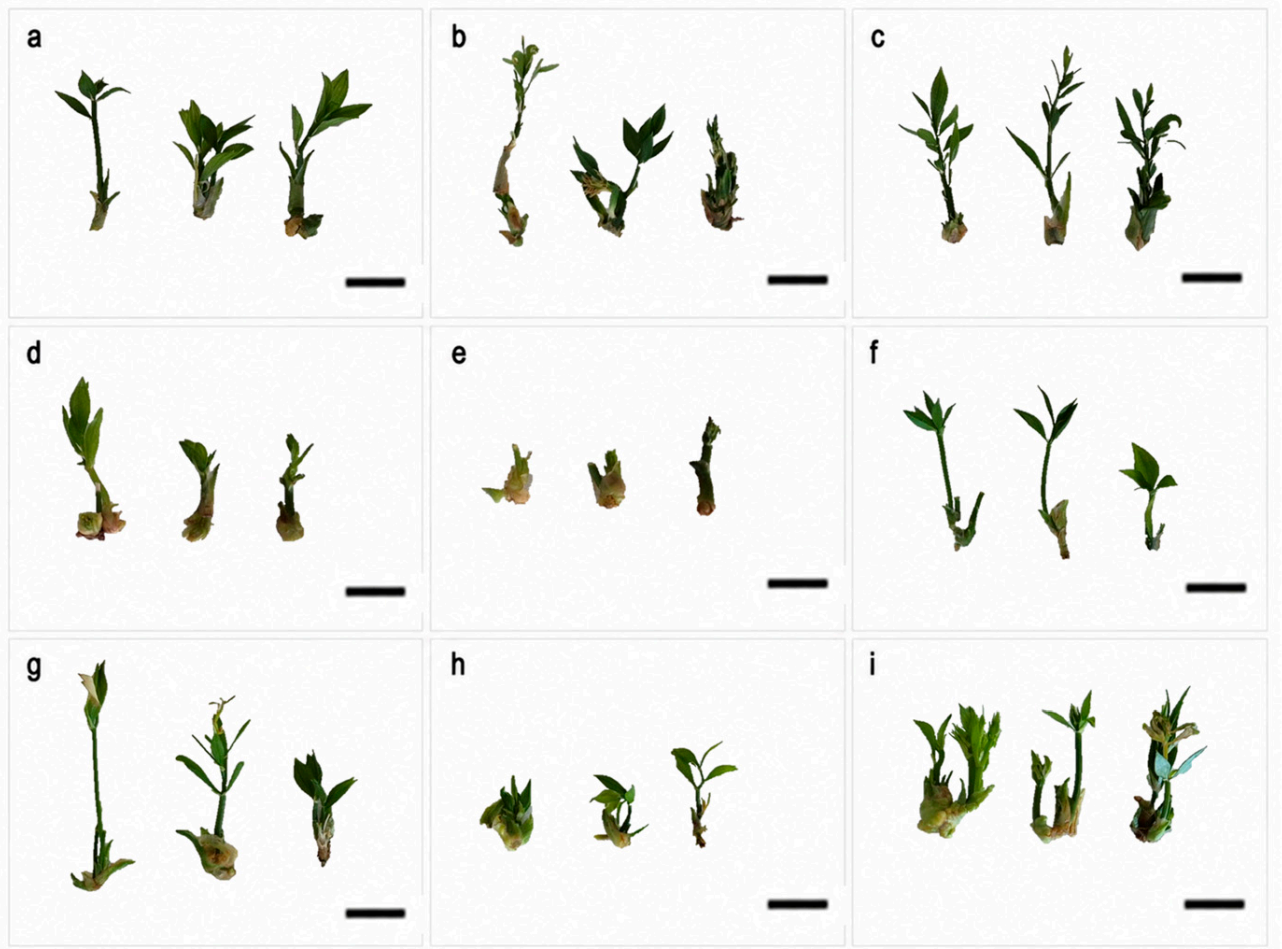
Representative in vitro morphological responses of *R. hypoglossum* rhizome explants to different cytokinin and spermidine treatments: (**a**) control; (**b**) 0.5 mg L^−1^ spermidine; (**c**) 1.0 mg L^−1^ spermidine; (**d**) 0.5 mg L^−1^ thidiazuron; (**e**) 1.0 mg L^−1^ thidiazuron; (**f**) 0.5 mg L^−1^ zeatin; (**g**) 1.0 mg L^−1^ zeatin; (**h**) 0.5 mg L^−1^ kinetin; and (**i**) 1.0 mg L^−1^ kinetin. Scale bars = 0.5 cm.

The effects of exogenous cytokinin and spermidine treatments on the morphological characteristics of *Ruscus hypoglossum* rhizome explants are presented in Figure 1 and Table 1. Significant treatment effects were observed for shoot number, cladode number, and fresh weight, whereas explant length and shoot diameter were not significantly affected. Among the treatments, 1.0 mg L^−1^ kinetin (K2) produced the highest shoot number (4.66 explant^−1^), cladode number (12.00 explant^−1^), and fresh weight (0.31 g), with values significantly exceeding those of all other treatments (*p* < 0.05). Furthermore, 1.0 mg L^−1^ zeatin (Z2) resulted in intermediate values for shoot number (3.00 explant^−1^) and cladode number (8.33 explant^−1^), whereas 1.0 mg L^−1^ thidiazuron (T2) produced the lowest cladode number (1.00 explant^−1^). The spermidine treatments exhibited a different response pattern. S2 (1.0 mg L^−1^ spermidine) produced the greatest numerical explant length (4.30 cm) and a relatively high cladode number (9.67 explant^−1^), whereas shoot number remained low (1.33 explant^−1^). However, differences in explant length among treatments were not statistically significant (*p* = 0.133).

### 2.2. Photosynthetic Pigments and SPAD

The effects of cytokinin and spermidine treatments on chlorophyll a, chlorophyll b, SPAD values, and total carotenoid content are presented in Table 2. All measured pigment-related parameters were significantly affected by the treatments (*p* ≤ 0.0063). Among the treatments, 1.0 mg L^−1^ kinetin (K2) produced the highest chlorophyll a (0.72 mg g^−1^ FW), chlorophyll b (0.29 mg g^−1^ FW), and SPAD values (40.25). In contrast, 1.0 mg L^−1^ thidiazuron (T2) resulted in the lowest chlorophyll a (0.06 mg g^−1^ FW), chlorophyll b (0.03 mg g^−1^ FW), SPAD value (25.88), and total carotenoid content (0.08 mg g^−1^ FW). The highest total carotenoid content was recorded in 1.0 mg L^−1^ spermidine (S2) (0.59 mg g^−1^ FW). This treatment also produced relatively high chlorophyll a (0.60 mg g^−1^ FW), chlorophyll b (0.25 mg g^−1^ FW), and SPAD values (36.01), whereas intermediate pigment levels were generally observed under the remaining treatments.

### 2.3. Multivariate Relationships Among Treatments and Morphophysiological Traits

**Figure 2:**
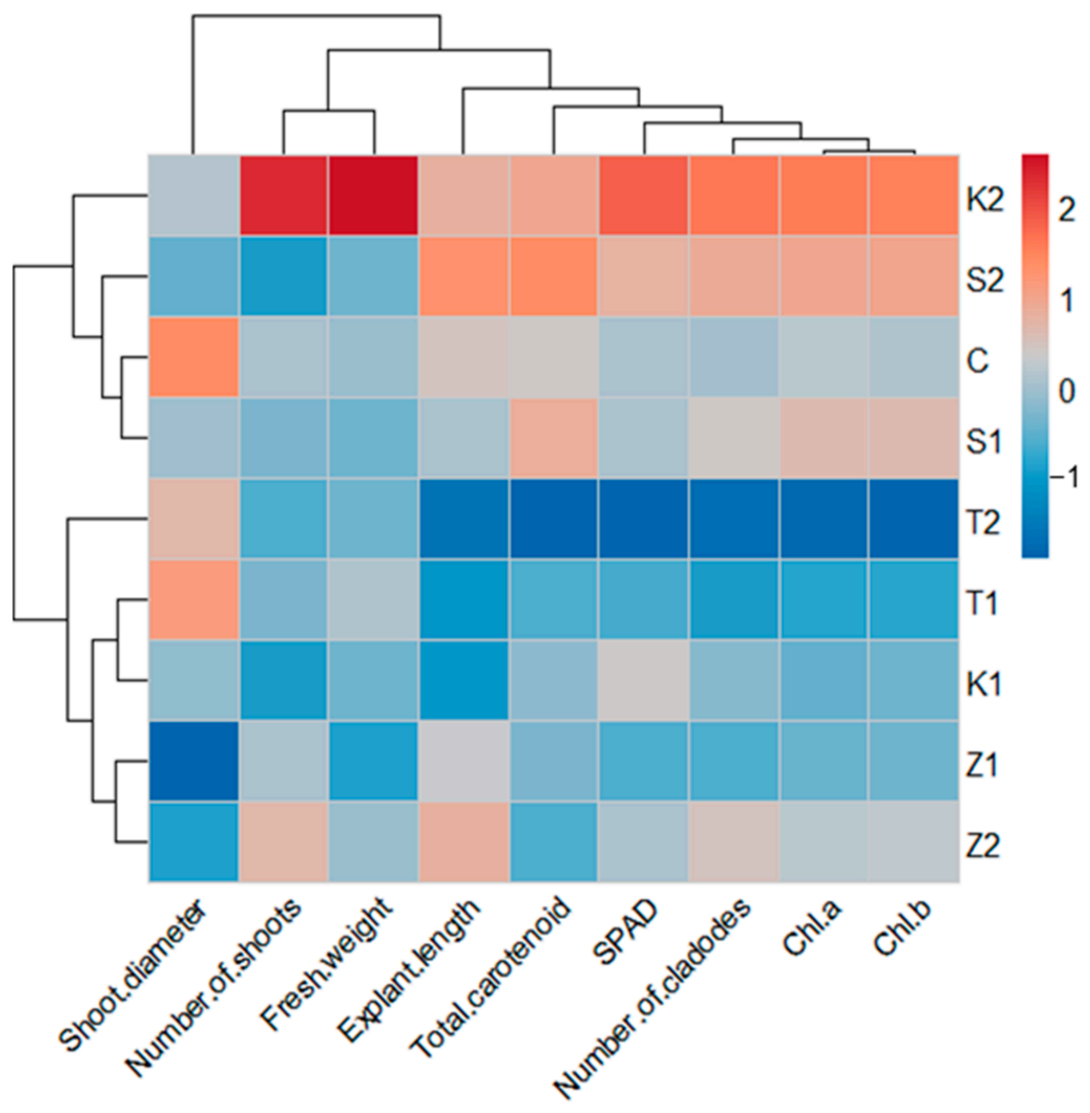
Heatmap of standardized morphological and pigment traits of in vitro-grown responses of *R. hypoglossum* explants subjected to cytokinin and spermidine treatments.

Hierarchical clustering of the standardized trait means revealed distinct treatment-specific morphophysiological response patterns (Figure 2). Red and blue tone colours indicate values above and below the overall trait mean, respectively. Dendrograms show hierarchical clustering of treatments and measured traits. K2 formed a clearly distinct cluster and was associated with high standardized values for shoot number, fresh weight, cladode number, SPAD, chlorophyll a, and chlorophyll b, indicating a strong overall response in both multiplication and physiological performance. S2 was primarily characterized by greater explant length and total carotenoid content, together with relatively favorable pigment values; however, it showed a weaker shoot proliferation response than K2, whereas the control was associated with a comparatively high standardized value for shoot diameter. Conversely, treatments T1 and, particularly, T2, exhibited low standardized values for most morphological and pigment-related traits. Trait clustering grouped shoot number with fresh weight, while SPAD, chlorophyll a, chlorophyll b, and the cladode number formed a separate cluster. Shoot diameter clustered independently from the remaining variables.

**Figure 3:**
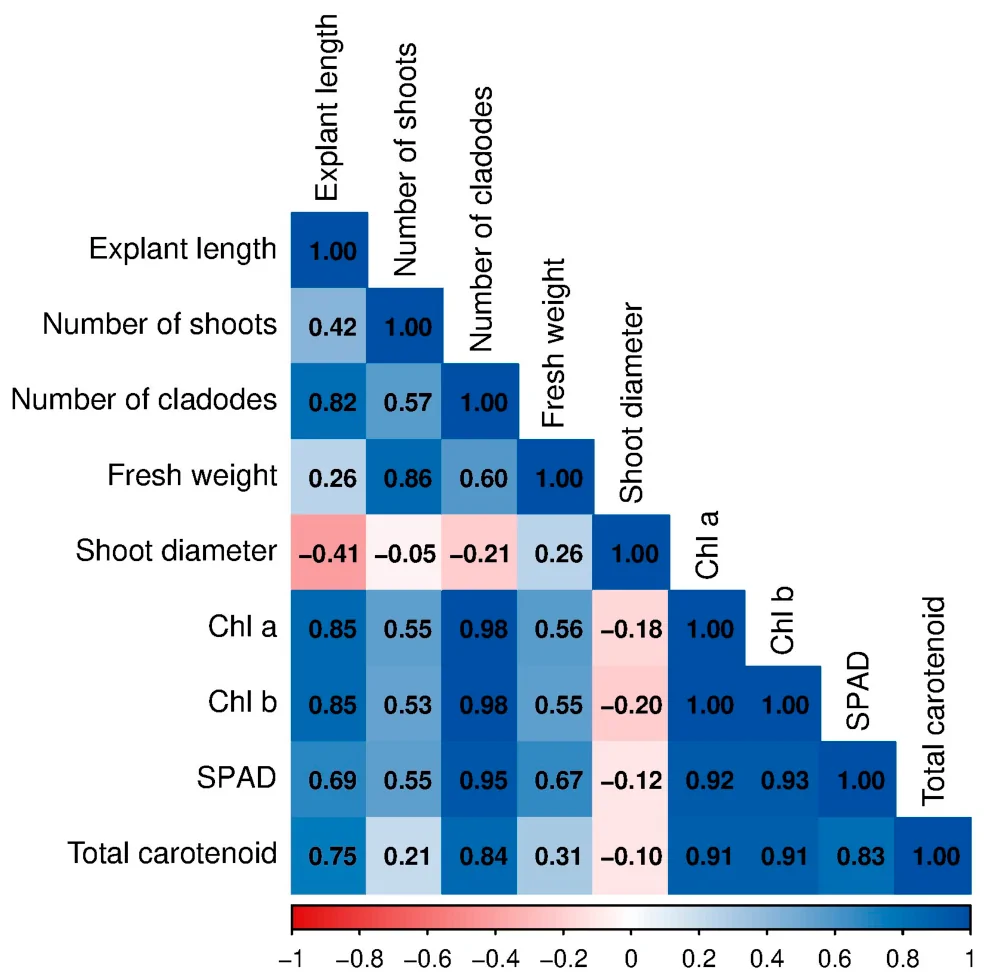
Pearson correlation matrix of morphological and physiological traits of in vitro-grown *R. hypoglossum* explants subjected to cytokinin and spermidine treatments. Values within the cells represent Pearson correlation coefficients.

Pearson correlation analysis showed strong relationships among the measured morphological and physiological traits (Figure 3). Cladode number was positively correlated with chlorophyll a (r = 0.98), chlorophyll b (r = 0.98), SPAD (r = 0.95), total carotenoids (r = 0.84), and explant length (r = 0.82). Shoot number showed the strongest positive correlation with fresh weight (r = 0.86). Chlorophyll a and chlorophyll b were strongly correlated (r = 1.00) and were also positively associated with SPAD (r = 0.92–0.93) and total carotenoids (r = 0.91). Explant length was positively correlated with chlorophyll a and chlorophyll b (r = 0.85 for both), whereas shoot diameter showed weak or negative correlations with most of the remaining variables, including explant length (r = −0.41).

**Figure 4:**
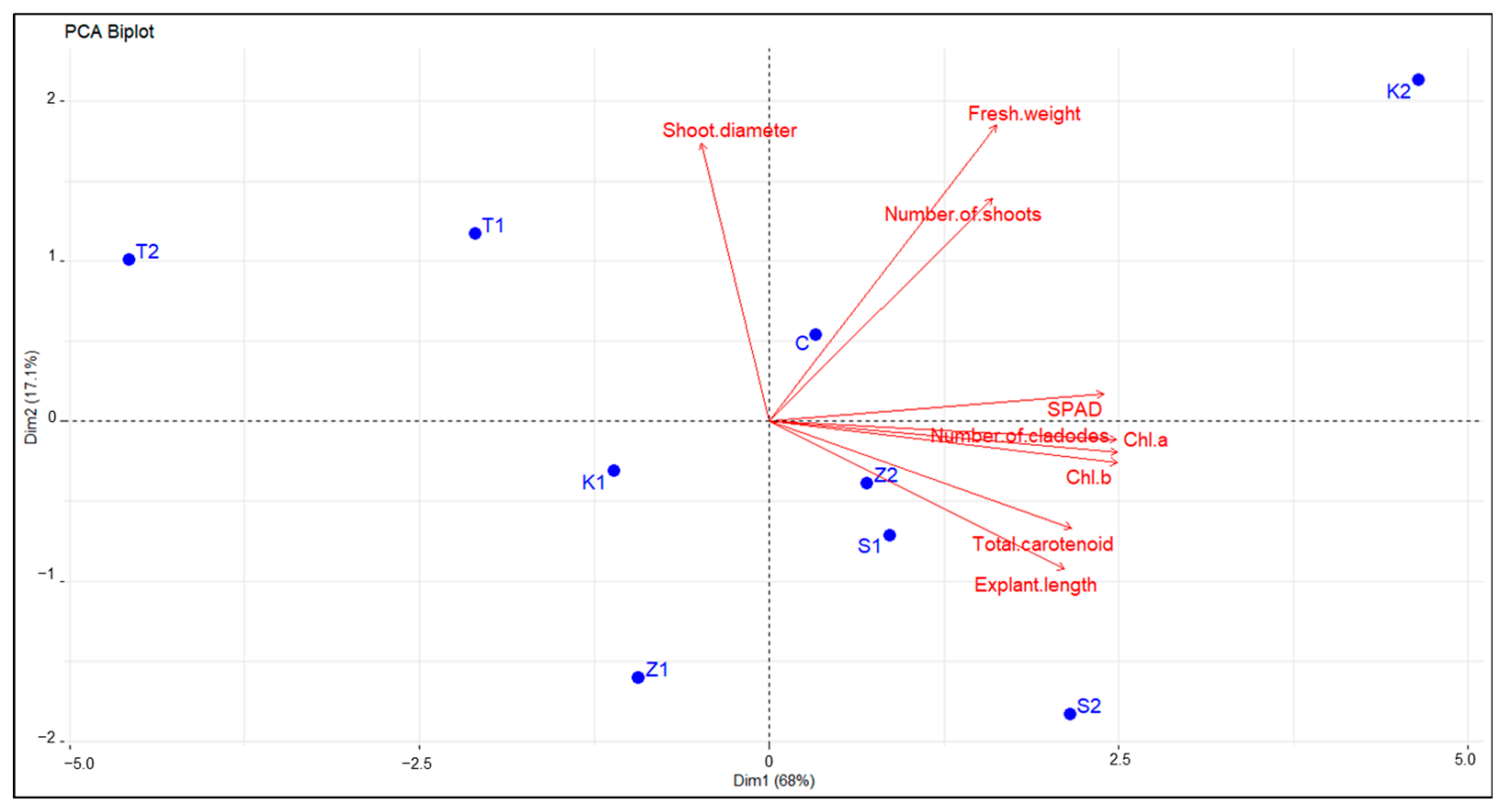
Principal component analysis biplot of morphological and pigment traits in *R. hypoglossum* explants under cytokinin and spermidine treatments. PC1 and PC2 explained 68.0% and 17.1% of the total variation, respectively.

Principal component analysis explained 85.1% of the total variation, with PC1 and PC2 accounting for 68.0% and 17.1%, respectively (Figure 4). Positive PC1 loadings were associated with shoot number, cladode number, fresh weight, chlorophyll a, chlorophyll b, SPAD, total carotenoids, and explant length. K2 was positioned on the positive side of PC1, whereas T1 and T2 were located on the negative side. S2 was positioned on the positive side of PC1 and the negative side of PC2 and was located closer to the vectors representing explant length and total carotenoids. The control treatment was positioned closer to the shoot diameter vector.

### 2.4. Effect of Previous Regeneration Medium and IBA Concentration on Rooting

**Figure 5:**
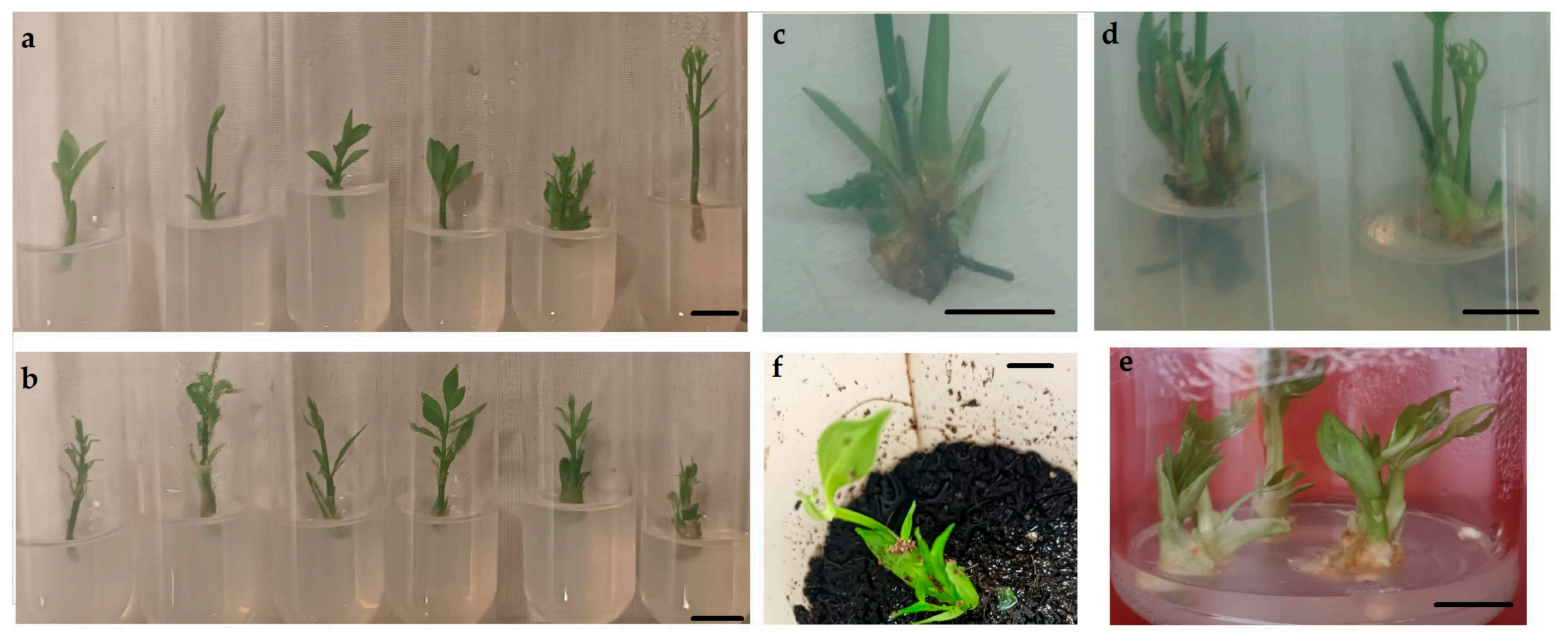
Rooting stages of *R. hypoglossum*: (**a**) Shoots derived from regenerated on 1.0 mg L^−1^ kinetin; (**b**) shoots regenerated on derived from 1.0 mg L^−1^ spermidine; (**c**) initial root emergence; formation; (**d**) root development after six weeks; (**e**) basal callus formation after eight weeks in explants not treated with IBA; (**f**) transfer of a rooted plantlet to peat (Scale bars = 1 cm).

Shoots regenerated on media supplemented with 1.0 mg L^−1^ kinetin or 1.0 mg L^−1^ spermidine exhibited sufficient development for transfer to the rooting stage (Figure 5a,b). Kinetin-derived shoots were generally more elongated, whereas spermidine-derived shoots were comparatively compact and produced multiple basal shoots. Following IBA treatment, the explants were transferred to IBA-free half-strength MS medium for root development. The first roots emerged from the basal region during the first week after transfer (Figure 5c), and well-developed rooted shoots were obtained after six weeks (Figure 5d). In contrast, callus formation was observed after eight weeks in explants that had not received IBA treatment (Figure 5e). Rooted plantlets were successfully transferred to ex vitro conditions and showed continued vegetative development during acclimatization (Figure 5f). However, acclimatization was performed as a qualitative confirmation of successful plant establishment, and quantitative survival data were not systematically recorded; therefore, survival percentage and statistical analysis could not be reported.

Rooting responses differed significantly among the combinations of previous regeneration media and IBA concentrations (Table 3).

Rooting responses varied according to the previous regeneration treatment and IBA pulse concentration (Table 3). Shoots regenerated on 1.0 mg L^−1^ kinetin showed the strongest overall rooting response following the 2.0 mg L^−1^ IBA pulse, whereas spermidine-derived shoots exhibited their highest root production at 1.0 mg L^−1^ IBA. Root length showed a comparatively similar response between the two higher IBA concentrations within each regeneration background. Overall, these results indicate that the physiological state established during the multiplication stage may influence subsequent responsiveness to IBA during root induction.

## 3. Discussion

The present study demonstrated distinct responses of *R. hypoglossum* rhizome explants to the tested cytokines and spermidine. Among the treatments evaluated, 1.0 mg L^−1^ kinetin produced the most favorable overall multiplication response, with higher shoot and cladode numbers and fresh weight, together with comparatively high pigment and SPAD values. The shoot diameter, however, was not significantly affected by the treatments. This finding indicates that the observed differences among treatments were primarily associated with shoot multiplication, cladode formation, and biomass accumulation rather than shoot thickness.

Previous studies indicate that the response of *Ruscus* to cytokinins is strongly influenced by the regeneration stage, explant type, and accompanying PGR regime. In *R. hypophyllum*, Ziv [18] reported a 6–10-fold increase in axillary and adventitious shoot proliferation after shoot-tip and inflorescence explants were initially cultured in agitated Linsmaier and Skoog liquid medium containing 1.0 mg L^−1^ BAP together with 1.0 mg L^−1^ 2,4-D, followed by transfer after 15 days to a solid medium with a higher cytokin-in-to-auxin ratio (5:1). Winarto and Setyawati [22] similarly demonstrated that regeneration and multiplication responses varied markedly with culture medium and PGR composition in *R. hypophyllum*. During the multiplication stage, the highest shoot production (9.2 shoots explant^−1^) was obtained on hormone-free half-strength MS medium, whereas three-quarter-strength NWT medium containing 0.25 mg L^−1^ BA, 0.5 mg L^−1^ kinetin, and 0.05 mg L^−1^ IAA produced 7.5 shoots explant^−1^. In *R. hypoglossum*, Abou-Dahab et al. [24] used full-strength MS medium supplemented with 1.5 mg L^−1^ BA and 2.0 mg L^−1^ kinetin as the cytokinin-containing basal regeneration medium. In contrast to these protocols, which involved cytokinin combinations or sequential changes in PGR composition, the present study showed that 1.0 mg L^−1^ kinetin alone supported a favorable combination of shoot multiplication and physiological performance in rhizome-derived explants. These differences among studies further emphasize that cytokinin responsiveness in *Ruscus* depends on species, explant source, regeneration pathway, PGR regime, and culture conditions.

Spermidine elicited a response that was distinct from that of kinetin. The 1.0 mg L^−1^ spermidine treatment demonstrated the most significant numerical increase in explant length and the highest carotenoid content, while shoot proliferation remained comparatively limited. Similar positive effects of spermidine on in vitro growth or regeneration-related traits have been reported in other plant species [32,33,34,35]. However, in the present study, spermidine did not produce a shoot multiplication response comparable to that obtained with 1.0 mg L^−1^ kinetin. The findings suggest that spermidine and kinetin induce divergent morpho-physiological responses under the evaluated conditions, excluding the implication of a particular hormonal or molecular mechanism.

TDZ treatments were associated with comparatively poor performance, particularly at 1.0 mg L^−1^, which resulted in low cladode formation and reduced chlorophyll, carotenoid, and SPAD values. This finding contrasts with the observations reported by Ivanova et al. [23], who documented the promotion of shoot development in *R. hypoglossum* when exposed to 0.5 mg L^−1^ TDZ. This variation underscores the notion that responses to plant growth regulators can vary depending on the explant material and the culture conditions. In the present study, the 1.0 mg L^−1^ treatment produced a poorer overall response than the 0.5 mg L^−1^ treatment within the two TDZ concentrations tested. It is imperative to acknowledge the limitations of the study, which evaluated only two concentrations.

The application of 1.0 mg L^−1^ of zeatin resulted in an intermediate multiplication response and increased cladode formation when compared to lower concentrations. However, this response remained less effective than the 1.0 mg L^−1^ kinetin for the primary multiplication traits.

The multivariate analyses were consistent with the univariate results and provided an integrated representation of the measured traits. K2 was associated with shoot number, cladode number, fresh weight, chlorophyll content, and SPAD, whereas S2 was more closely associated with carotenoid content and numerical explant elongation. TDZ treatments exhibited comparatively low values for several morphological and pigment-related traits. Therefore, these analyses support the differentiation of treatment-specific response profiles. However, they are not interpreted as evidence of underlying molecular or hormonal mechanisms.

Furthermore, the responses of the roots to rooting also exhibited variation according to the regeneration treatment from which the shoots originated and the IBA concentration used during root induction. Shoots obtained from 1.0 mg L^−1^ kinetin demonstrated the highest rooting percentage and root number when transferred to half-strength MS medium containing 2.0 mg L^−1^ IBA, whereas shoots obtained from the spermidine treatment produced their highest root number at 1.0 mg L^−1^ IBA. Concurrent studies have reported conflicting results regarding the rooting requirements of *Ruscus* propagation systems. Purwito et al. [20] obtained rooting of *R. hypophyllum* on half-strength MS medium containing 2.0 mg L^−1^ IBA. Conversely, Jha and Sen [19] and Winarto and Setyawati [22] reported successful rooting on plant-growth-regulator-free MS or half-strength MS media. The present results therefore indicate that both the regeneration background and IBA treatment should be considered when developing a rooting procedure for *R. hypoglossum*.

In summary, the present study identifies 1.0 mg L^−1^ kinetin as the most effective treatment among those evaluated for shoot multiplication and subsequent rooting of *R. hypoglossum*. However, it is imperative to acknowledge the limitations of the study, which are primarily constrained by the tested plant material, the concentrations of the regulators, and the culture conditions. Further validation is required across a range of additional genotypes and independent experimental cycles before the protocol can be widely applied.

## 4. Materials and Methods

### 4.1. Plant Material and Establishment of Seed-Origin Cultures

Mature fruits of *Ruscus hypoglossum* were collected in 2024 from a natural population in Arhavi, Artvin, Türkiye (41.289800° N, 41.294630° E; 236 m a.s.l.). The seeds were meticulously extracted from the fruit pulp, thoroughly rinsed under running tap water, dried on a sterile surface, and stored in glass containers at ambient temperature in darkness until utilization.

The seed germination protocol established by Tütüncü et al. [25] for *R. hypoglossum* was used as the methodological basis for the present study. The seeds were subsequently transferred to 500 mL of distilled water and exposed to ozone for 30 min using a corona-discharge ozone generator with a nominal output capacity of 6 g h^−1^.

The seeds were surface-sterilized in 70% ethanol for 1–2 min, followed by 25% commercial bleach solution (Domestos^®^, 4.5% *v*/*v*) for 15 min, and then rinsed four times with sterile distilled water. The disinfected seeds were then cultivated in Petri dishes containing 25 mL of full-strength Murashige and Skoog (MS) [36] medium, which had been supplemented with 30 g L^−1^ sucrose and solidified with 7 g L^−1^ agar. Murashige and Skoog basal medium including vitamins was obtained from Duchefa Biochemie B.V. (M0222; Haarlem, The Netherlands). The culture media were solidified using Bacto™ Agar (catalog no. 214010; BD, Sparks, MD, USA).

The medium pH was adjusted to a range of 5.6–5.8 prior to autoclaving at 121 °C for a duration of 20 min. Cultures were subjected to an incubation process at a temperature of 23 ± 2 °C, in conditions of darkness, for a period of two months. Thereafter, subculturing was performed at four-week intervals. After the sixth subculture cycle, plantlets that had reached a uniform and sufficiently developed growth stage were selected for the experiment, and rhizome segments excised from these plantlets were used as explants.

### 4.2. Regulator Treatments and Culture Conditions

Rhizome explants of uniform size were aseptically excised from well-established in vitro plants and inoculated on full-strength MS medium containing 30 g L^−1^ sucrose and 7 g L^−1^ agar. Nine treatments were evaluated: a regulator-free control; spermidine, TDZ, zeatin, and kinetin, each at 0.5 and 1.0 mg L^−1^ (Table 4).

The medium’s pH was adjusted to 5.7 ± 0.1 prior to autoclaving at 121 °C for 20 min. Heat-labile supplements, when applicable, were filter-sterilized and added to cooled autoclaved medium. Cultures were maintained at a temperature of 24 ± 1 °C under cool-white fluorescent lamps (PPFD: 40 μmol m^−2^ s^−1^, 16-h photoperiod. Each treatment comprised three biological replicates, with 10 explants per replicate. Each 25 × 150 mm culture tube (Pyrex^®^, Corning Inc., Corning, NY, USA) contained a single rhizome explant. Subculturing of cultures was undertaken at five-week intervals, and a comprehensive set of morphological and physiological measurements were systematically recorded following two consecutive subculture cycles. This equates to a total culture period of 10 weeks.

### 4.3. Morphological Measurements

At the end of the culture period, the following parameters were recorded: explant length, number of shoots per explant, number of cladodes per explant, fresh weight, and shoot diameter. The length of the explant was measured from the basal end to the apical extremity of the longest shoot and expressed in cm. The shoot diameter was measured at the basal portion of the main shoot using a digital caliper and expressed in mm. The weight of the explants was determined immediately after their removal from the culture vessels, with the residual medium being meticulously removed.

### 4.4. Photosynthetic Pigment Measurements

Chlorophyll a, chlorophyll b, total carotenoids, and SPAD values were determined from fully expanded, healthy cladodes.

Chlorophyll status was estimated non-destructively using a SPAD-502Plus chlorophyll meter (Konica Minolta, Tokyo, Japan). Measurements were taken from fully expanded, visually healthy cladodes. Three SPAD readings were taken from the central region of each fully expanded cladode, and the mean was used as the plant-level value.

Chlorophyll a, chlorophyll b, and total carotenoids were determined spectrophotometrically. Spectrophotometric quantification of chlorophylls and carotenoids was performed using a Shimadzu UV-1800 spectrophotometer (Shimadzu Corporation, Kyoto, Japan) equipped with 1 cm quartz cuvettes, with the instrument calibrated against the corresponding blank before each measurement. Fresh cladode tissue (0.10 g) was homogenized in 10 mL of 80% (*v*/*v*) acetone under dim-light conditions. The homogenate was centrifuged at 10,000× *g* for 10 min, and absorbance of the clear supernatant was measured at 663, 645, and 470 nm using 80% acetone as the blank. Chlorophyll a and chlorophyll b concentrations were calculated according to Arnon [37], whereas total carotenoids were calculated according to Lichtenthaler and Wellburn [38]:

Chl a = 12.7(A_663_) − 2.69(A_645_); (1)

Chl b = 22.9(A_645_) − 4.68(A_663_); (2)

Total carotenoids = [1000(A_470_) − 1.82(Chl a) − 85.02(Chl b)]/198 (3)

where *A*_663_, *A*_645_, and *A*_470_ represent the absorbance values measured at 663, 645, and 470 nm, respectively. Pigment concentrations calculated using these equations were expressed as µg mL^−1^ of extract. Pigment contents were subsequently converted to mg g^−1^ fresh weight (FW) using the following equation:

Pigment content (mg g^−1^ FW) = (*C × V*)/(1000 × *W*) (4)

where *C* represents the pigment concentration in the extract (µg mL^−1^), *V* is the final extract volume (10 mL), and *W* is the fresh weight of the sample (0.10 g).

### 4.5. Root Induction

Based on the regeneration-stage responses, shoots cultured on MS medium supplemented with 1.0 mg L^−1^ kinetin and 1.0 mg L^−1^ spermidine were selected for the rooting experiment. Kinetin was chosen because it produced the most favorable overall multiplication response, including higher shoot proliferation, cladode formation, and photosynthetic performance, whereas spermidine was selected because it promoted greater shoot elongation and physiological quality despite its comparatively lower multiplication rate. In addition, shoots from both treatments had attained sufficient length and morphological development for transfer to the rooting stage, allowing the rooting competence of two contrasting but well-performing regeneration treatments to be compared. Three IBA concentrations were evaluated: 0, 1.0, and 2.0 mg L^−1^. Indole-3-butyric acid (IBA; I5386; BioReagent, suitable for plant cell culture) was obtained from Sigma-Aldrich (St. Louis, MO, USA). The IBA solutions were sterilized through a 0.22 µm membrane filter and dispensed into sterile microcentrifuge tubes with the caps removed. The basal ends of the shoots were immersed in the corresponding IBA solution (0, 1.0, or 2.0 mg L^−1^) for 10 s and then transferred to half-strength MS medium (containing 20 g L^−1^ sucrose and 6.5 g L^−1^ agar).

Each treatment consisted of at least three biological replicates, with nine explants per replicate. Each 25 × 150 mm culture tube contained a single explant. The cultures were maintained for 10 weeks under the same growth-room conditions described previously. At the end of the rooting period, rooting percentage, number of roots per explant, and root length were recorded.

### 4.6. Experimental Design and Statistical Analysis

The shoot regeneration experiment was arranged in a completely randomized design with nine treatments and three biological replicates per treatment. Data were subjected to one-way analysis of variance (ANOVA), with treatment included as the fixed factor. When the ANOVA indicated a significant treatment effect, means were separated using Tukey’s honestly significant difference (HSD) test at α = 0.05. Individual observations within each treatment were used for the statistical analyses, and treatment mean values were calculated and presented in the corresponding tables. Statistical analyses were performed using JMP Pro 17 (SAS Institute Inc., Cary, NC, USA). To examine relationships among the measured morphological and physiological traits, Pearson correlation analysis, principal component analysis (PCA), and hierarchical clustering with heatmap visualization were conducted. Before multivariate analyses, all variables were standardized using Z-scores. The analyses and graphical visualizations were performed in R version 4.5.2 using the FactoMineR, factoextra, corrplot, and heatmap packages.

## 5. Conclusions and Future Perspectives

The present study demonstrated distinct morpho-physiological responses of *Ruscus hypoglossum* rhizome explants to the tested growth regulators. Among the treatments evaluated, 1.0 mg L^−1^ kinetin produced the most favorable overall multiplication response, particularly in terms of shoot proliferation, cladode formation, biomass accumulation, and pigment-related traits. Spermidine produced a contrasting response, characterized mainly by greater explant elongation and carotenoid content. In contrast, the higher TDZ concentration resulted in comparatively poor performance. The responses to rooting exhibited variability in accordance with the regeneration treatment and IBA concentration. Notably, kinetin-derived shoots demonstrated the highest rooting percentage at 2.0 mg L^−1^ IBA. In sum, these findings provide a solid foundation for the subsequent refinement of an in vitro propagation protocol for *R. hypoglossum* under the examined culture conditions.

It is imperative that future studies employ additional genotypes and independent experimental cycles to validate these findings. Furthermore, the acclimatization stage should be optimized through quantitative evaluation of ex vitro survival and subsequent plant performance. The assessment of genetic fidelity and greenhouse or nursery performance of regenerated plants would also serve to strengthen the practical applicability of the protocol. Furthermore, the efficacy of broader concentration ranges and sequential or combined applications of cytokines and polyamines in enhancing shoot multiplication and rooting responses warrants further investigation. Subsequent analyses, whether molecular, hormonal, or biochemical, could provide further insights by investigating the biological underpinnings of the treatment-specific responses observed in *R. hypoglossum*.

## Figures and Tables

**Table 1:** Effects of cytokinin and spermidine treatments on in vitro morphological traits of *R. hypoglossum*.

Treatment	Explant Length (cm)	Number of Shoots (n)	Number of Cladodes (n)	Fresh Weight (g)	Shoot Diameter (mm)
C	3.70	2.33 bc	6.66 bcd	0.15 b	2.31
S1	3.40	2.00 bc	8.00 abc	0.13 b	1.75
S2	4.30	1.33 c	9.67 ab	0.13 b	1.57
T1	2.60	2.00 bc	3.66 de	0.16 b	2.20
T2	2.23	1.66 c	1.00 e	0.13 b	2.02
Z1	3.60	2.33 bc	4.67 cde	0.10 b	1.04
Z2	3.93	3.00 b	8.33 abc	0.15 b	1.42
K1	2.63	1.33 c	6.00 bcd	0.13 b	1.71
K2	3.93	4.66 a	12.00 a	0.31 a	1.82
*p*-value	0.133	0.0003	0.0016	0.0062	0.083
Significance	ns	***	**	**	ns

Values represent treatment means. Different lowercase letters within the same column indicate significant differences among treatments according to Tukey’s HSD test at *p* < 0.05; ns, not significant; ** *p* < 0.01; *** *p* < 0.001.

**Table 2:** Effects of cytokinin and spermidine treatments on photosynthetic pigments and SPAD values of in vitro-grown *R. hypoglossum* explants.

Treatment	Chl a (mg g^−1^ FW)	Chl b (mg g^−1^ FW)	SPAD	Total Carotenoid (mg g^−1^ FW)
C	0.45 bcd	0.18 bcd	33.28 c	0.44 abc
S1	0.54 abc	0.22 abc	33.23 cd	0.51 ab
S2	0.60 ab	0.25 ab	36.01 b	0.59 a
T1	0.25 de	0.11 de	30.36 e	0.28 cd
T2	0.06 e	0.03 e	25.88 f	0.08 d
Z1	0.33 cd	0.14 cd	30.65 de	0.33 bc
Z2	0.45 bcd	0.19 bcd	33.23 cd	0.28 abc
K1	0.32 cd	0.14 cd	34.42 bc	0.35 bc
K2	0.72 a	0.29 a	40.25 a	0.52 ab
*p*-value	0.0006	0.0007	<0.0001	0.0063
Significance	***	***	***	**

Values represent treatment means. Different lowercase letters within the same column indicate significant differences among treatments according to Tukey’s HSD test at *p* < 0.05; ** *p* < 0.01; *** *p* < 0.001.

**Table 3:** Rooting responses of *Ruscus hypoglossum* shoots as affected by previous regeneration media and IBA concentrations.

Previous Regeneration Medium	IBA (mg L^−1^)	Rooting Percentage (%)	Number of Roots per Explant (n)	Root Length (cm)
1.0 mg L^−1^ Kinetin	0	7.41 d	0.67 b	0.37 c
	1	18.52 bc	1.33 b	1.59 a
	2.0	44.44 a	4.00 a	1.50 a
1.0 mg L^−1^ Spermidine	0	18.52 c	1.00 b	0.38 c
	1	25.93 b	3.48 a	0.73 bc
	2.0	25.93 b	1.00 b	1.03 b
*p*-value		<0.0001	0.0002	0.0016
Significance		***	***	**

Values represent treatment means based on 27 shoots per treatment (n = 27). Rooting percentage was calculated as the proportion of shoots that formed roots. Different lowercase letters within the same column indicate significant differences among treatments according to Tukey’s HSD test at *p* < 0.05. ** *p* < 0.01; *** *p* < 0.001.

**Table 4:** Plant growth regulator and polyamine treatments used for rhizome-derived regeneration of *R. hypoglossum*.

Code	Compound Class	Supplement	Concentration (mg L^−1^)
C	Control	None	0
S1	Polyamine	Spermidine *	0.5
S2	Polyamine	Spermidine *	1.0
T1	Phenyl urea cytokinin-like regulator	Thidiazuron *	0.5
T2	Phenyl urea cytokinin-like regulator	Thidiazuron *	1.0
Z1	Isoprenoid cytokinin	Zeatin *	0.5
Z2	Isoprenoid cytokinin	Zeatin *	1.0
K1	Purine cytokinin	Kinetin *	0.5
K2	Purine cytokinin	Kinetin *	1.0

* Kinetin (K0753), zeatin (Z0164), thidiazuron (P6186), and spermidine (85558; BioUltra, ≥99.5% purity) were obtained from Sigma-Aldrich (St. Louis, MO, USA).

## Data Availability

The data presented in this study are available within the article. The data supporting the findings of this study are available from the corresponding author upon reasonable request.

## Abbreviations

The following abbreviations are used in this manuscript:

TDZ	N-Phenyl-N’-1,2,3-thiadiazol-5-ylurea, Thidiazuron
SPAD	Soil–Plant Analysis Development
MS	Murashige and Skoog medium
IBA	Indole-3-butyric acid
IAA	Indole-3-acetic acid
NAA	1-Naphthaleneacetic acid
BAP	6-Benzylaminopurine
BA	Benzyl adenine
2,4-D	2,4-Dichlorophenoxyacetic acid
FW	Fresh weight
PCA	Principal Component Analysis
